# Milk Consumption Across Life Periods in Relation to Lower Risk of Nasopharyngeal Carcinoma: A Multicentre Case-Control Study

**DOI:** 10.3389/fonc.2019.00253

**Published:** 2019-04-10

**Authors:** Zhi-Ming Mai, Jia-Huang Lin, Roger Kai-Cheong Ngan, Dora Lai-Wan Kwong, Wai-Tong Ng, Alice Wan-Ying Ng, Kam-Tong Yuen, Dennis Kai Ming Ip, Yap-Hang Chan, Anne Wing-Mui Lee, Sai-Yin Ho, Maria Li Lung, Tai-Hing Lam

**Affiliations:** ^1^School of Public Health, The University of Hong Kong, Hong Kong, China; ^2^Centre for Nasopharyngeal Carcinoma Research (CNPCR), Research Grants Council Area of Excellence Scheme, The University of Hong Kong, Hong Kong, China; ^3^Department of Clinical Oncology, Queen Elizabeth Hospital, Hong Kong, China; ^4^Department of Clinical Oncology, The University of Hong Kong, Hong Kong, China; ^5^Department of Clinical Oncology, Pamela Youde Nethersole Eastern Hospital, Hong Kong, China; ^6^Department of Clinical Oncology, Tuen Mun Hospital, Hong Kong, China; ^7^Department of Oncology, Princess Margaret Hospital, Hong Kong, China; ^8^Department of Medicine, Queen Mary Hospital, The University of Hong Kong, Hong Kong, China; ^9^Clinical Oncology Center, The University of Hong Kong-Shenzhen Hospital, Shenzhen, China

**Keywords:** milk, nasopharyngeal carcinoma, case-control study, life-course, multiple imputation

## Abstract

**Background:** The much higher incidence of nasopharyngeal carcinoma (NPC) in men suggests sex hormones as a risk factor, and dairy products contain measurable amounts of steroid hormones. Milk consumption has greatly increased in endemic regions of NPC. We investigated the association between NPC and milk consumption across life periods in Hong Kong.

**Methods:** A multicentre case-control study included 815 histologically confirmed NPC incident cases and 1,502 controls who were frequency-matched on age and sex at five major hospitals in Hong Kong in 2014–2017. Odds ratios (ORs) of NPC (cases vs. controls) for milk consumption at different life periods were estimated by unconditional logistic regression, adjusting for sex, age, socioeconomic status score, smoking and alcohol drinking status, exposure to occupational hazards, family history of cancer, IgA against Epstein-Barr virus viral capsid antigen, and total energy intake.

**Results:** Compared with abstainers, lower risks of NPC were consistently observed in regular users (consuming ≥5 glasses of milk [fresh and powdered combined] per month) across four life periods of age 6–12 (adjusted OR 0.74, 95% CI 0.54–0.86), 13–18 (0.68, 0.55–0.84), 19–30 (0.68, 0.55–0.84), and 10 years before recruitment (0.72, 0.59–0.87). Long-term average milk consumption of ≤2.5, >2.5, and ≤12.5, >12.5 glasses per month yielded adjusted OR (95% CI) of 1.00 (0.80–1.26), 0.98 (0.81–1.18), 0.95 (0.76–1.18), and 0.55 (0.43–0.70), respectively (all *P*-values for trend < 0.05).

**Conclusion:** Consumption of milk across life periods was associated with lower risks of NPC. If confirmed to be causal, this has important implications for dairy product consumption and prevention of NPC.

## Background

The etiology of nasopharyngeal carcinoma (NPC) is unclear, and its male predominance has been linked to sex hormones (1). Dairy products are a source of steroid hormones (2) and contain numerous potential antitumor substances.

Using an ecological study design, we found that increasing consumption of dairy products might explain the declining NPC incidence in 48 countries/regions (3, 4). Six case-control studies in other countries/regions [Malaysia (5), Guangzhou (6), Shanghai (7), Taiwan (8), Italy (9), and Maghrebian countries (10)] had examined the association between dairy intake and NPC risk. Three of them measured consumption of milk and cow's milk, while the others measured milk drinking with daily meals, and intake of rancid butter, and milk, and yogurt. Results were mixed with positive, null and negative associations. Milk is not a major component of the East Asian traditional diet but consumption has greatly increased with economic growth and globalization (11). We conducted a multicentre NPC case-control study in Hong Kong to further examine such association.

## Methods

The methods of this case-control study have been detailed elsewhere (12). Briefly, the cases were 815 histologically and/or radiologically confirmed incident NPC patients (response rate 78.4%) recruited in 2014–2017 from five major regional hospitals that treat up to 70% of all NPC cases in Hong Kong. The controls were 1,502 frequency-matched (by 5-year age group and sex; response rate 85.1%) new patients or referrals of a new health complaint in the past 12 months in specialist outpatient clinics, or new inpatients admitted in the past 3 months in the same hospitals. Those with a history of NPC, dementia, or suspected symptoms of NPC such as recent unilateral facial nerve palsy, tinnitus, unilateral hearing loss and epistaxis were excluded. Following the AsiaLymph guideline of the US National Cancer Institute (13), we also specified that no more than 15% of controls had the same specific type of disease. A limited number of specific diagnoses were further excluded, based on a known or suspected relation with vitamin D exposure, and immunological, infectious and/or inflammatory etiology. The disease list of controls is shown in Supplementary Part I.

### Consumption of Milk and Other Dairy Products

The subjects reported their average monthly consumption of dairy products over four life periods (age 6–12, 13–18, and 19–30, and 10 years before recruitment for fresh and powered milk; age 13–18 and 19–30, and 10 years before recruitment for other dairy products) on a computer-assisted, self-administered questionnaire with satisfactory test-retest reliability (coefficients 0.4–0.8) (12). Dairy consumption was categorized as: (1) milk [fresh and powdered milk combined, in glasses (one glass = 250 ml)], and (2) other dairy products [ice cream, yogurt or cheese, in servings (one cup of ice cream, one cup of yogurt, or 50 g of firm cheese)]. Those who consumed <5 glasses/month of milk (fresh and powdered combined) or ≤8 servings/month of other dairy products (ice cream, yogurt or cheese) were classified as “non-regular users,” those who consumed ≥5 glasses/month of milk or >8 servings/month of other dairy products as “regular users,” and those who never consumed as “abstainers.” To overcome the limitations of conventional approaches by using self-reported exposure, rs4988235 was genotyped as an “instrumental variable” to “unbiasedly” assess the association between dairy intake and NPC risk. Genotyping for the LCT-13910 C/T (rs4988235) polymorphism (14) was conducted using iPLEX assay on the MassARRAY System (Sequenom, San Diego, CA, USA) in 512 NPC cases and 898 controls (data not shown because only one case and three controls had the T allele that was associated with lactase persistence).

### Covariates

We also collected information on sex, 5-year age group, socioeconomic status score [range: −1 (lowest) to 13 (highest), calculated by the subject's, and his/her father's and mother's education, housing type at age 10, personal income, and household income], smoking and drinking status, occupational hazards, family history of cancer, and total energy intake. Dietary information was collected with the Semi-Quantitative Food Frequency Questionnaire with about 30 food items (12). The subjects reported how often, on average, they consumed a specified portion size of each food during the preceding year. We calculated total energy intake (residual method) (15) by multiplying the frequency of consumption of each item by its caloric content and summing the products across all foods in a specific period using the China Food Composition Table (2008 No. 2). Our questionnaire had acceptable test-retest reliability (coefficients 0.4–0.8) (12).

Antibody of IgA against Epstein-Barr virus (EBV) viral capsid antigen (VCA-IgA) was measured using a commercial kit (EUROIMMUN AG, Lübeck, Germany) based on the standard method of ELISA. Results were evaluated semi-quantitatively by calculating the ratio of the optical density value of the sample over the optical density value of the calibrator, expressed as relative optical density (rOD). According to the manufacturer's instruction, the serostatus of VCA-IgA was classified as seronegative (rOD value: <1.2) or seropositive (rOD value: ≥1.2).

### Statistical Analysis

Case vs. control odds ratios (ORs) for dairy consumption (non-regular/regular users vs. abstainers) were calculated using unconditional logistic regression, with/without adjusting for potential confounders. Odds ratios were calculated for dairy consumption at each life period and as average values across all periods to represent long-term intake. The group-specific confidence interval (CI) for the abstainers' OR of 1.00 was calculated using Plummer's methods to reflect the variance of the log odds (16).

To assess dose-response effect, a test for linear trend was examined for each categorical exposure. Interaction by sex was tested based on the likelihood ratio test by introducing interaction terms into the crude model.

We predicted missing values of the exposure and confounders (EBV VCA-IgA serostatus: 296 cases/478 controls, smoking status: 6/5, family history of cancer: 124/111 and exposure to occupational hazards: 131/147) based on a flexible additive regression model with predictive mean matching incorporating data on the factors included in the multivariable model (17). As a sensitive analysis, we also conducted a complete case analysis (Supplementary Table 1, the results were similar to those with multiple imputation). Statistical analyses were done with R 3.5.1, and all tests were two-sided with α = 0.05.

## Results

Table 1 shows that the cases were older and had a greater proportion of men, lower socioeconomic status, family history of NPC, ever-smoking, EBV seropositivity, and exposure to any occupational hazards compared with the controls (all *P*-values < 0.001). No difference in alcohol drinking status was observed (*P* = 0.19).

**Table 1:** Characteristics of nasopharyngeal carcinoma (NPC) cases and controls in five regional hospitals in Hong Kong, China 2014–2017.

**Characteristics**	**NPC cases (*****N*** **=** **815)**		**Controls (*****N*** **=** **1502)**		***P*-value^‡^**
	***n***	**%**	***n***	**%**	
**Sex**					0.001
Men	613	75.2	1028	68.4	
**Age at recruitment, years**					
Mean (interquartile range)	52.6 (44–59)		51.5 (42–60)		0.05
18– <35	54	6.7	179	11.9	0.001
35– <45	146	17.9	241	16.1	
45– <55	234	28.6	391	26.1	
55– <65	267	32.6	448	29.9	
≥65	114	14.2	243	16.1	
**Socioeconomic status score 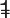 **					
Mean (SD)	3.0 (2.8)		3.7 (3.0)		< 0.001
**Family history of cancer**					< 0.001
None	288	35.3	753	50.1	
Had any family member(s) with history of cancer, excluding NPC	269	33.0	561	37.4	
Had any family member(s) with history of NPC	134	16.4	77	5.1	
Don't know	124	15.2	111	7.4	
**Exposure to any occupational hazards**					< 0.001
None	285	35.0	758	50.5	
Ever exposed	399	49.0	597	39.8	
Don't know	131	16.1	147	9.8	
**Smoking**					< 0.001
Never	417	51.2	945	62.9	
Ever	392	48.1	552	36.8	
Refuse to answer	6	0.7	5	0.3	
**Alcohol drinking**					0.19
Never	512	62.8	977	65.1	
≤210 g/week	203	24.9	377	25.1	
>210 g/week	100	12.3	148	9.9	
**EBV VCA-IgA^†^**					< 0.001
Seronegative	56	10.8	900	88.1	
Seropositive	463	89.2	123	11.9	

‡ *t-test and Chi-square test were used to compare the mean of continuous factors, and proportions of categorical factors between cases and controls, respectively*.

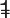
 *Socioeconomic status score ranged from−1 (lowest socioeconomic status) to 13 (highest socioeconomic status), and was calculated by the subject's, and his/her father's and mother's education, personal income, household income and housing type at age 10*.

† *Epstein-Barr virus viral capsid antibody (EBV VCA-IgA) levels: optical density value <1.2 (seronegative) or ≥1.2 (seropositive). We excluded subjects who had not provided blood, or whose plasma EBV VCA-IgA was not measured*.

Table 2 shows, compared with abstainers, the adjusted ORs (95% CI) of NPC in regular users who consumed ≥5 glasses of milk (fresh and powdered combined) per month were 0.74 (0.61–0.91) at age 6–12, 0.68 (0.54–0.86) at age 13–18, 0.68 (0.55–0.84) at age 19–30, and 0.72 (0.59–0.87) 10 years before recruitment (all *P*-values for trend <0.05). For other dairy products (ice cream, yogurt or cheese), compared with abstainers, the adjusted ORs (95% CI) in non-regular users who consumed ≤8 servings/month were 0.85 (0.74–0.97) at age 13–18, 0.84 (0.73–0.96) at age 19–30, and 0.88 (0.77–1.01) 10 years before recruitment, and in regular users who consumed >8 servings/month were 0.92 (0.74–1.14) at age 13–18, 1.01 (0.81–1.25) at age 19–30, and 1.05 (0.85–1.31) 10 years before recruitment (all *P*-values for trend >0.05).

**Table 2:** Odds ratios (ORs) and 95% confidence intervals (CI) of nasopharyngeal carcinoma for dairy product consumption in 815 NPC cases and 1,502 controls after multiple imputation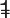.

		***N* cases/controls**	**Age- and sex-adjusted**	**Multivariable adjusted**
			**OR (95% CI)**	**OR^†^ (95% CI)**
**MILK (FRESH AND POWDERED COMBINED), GLASSES/MONTH**				
At age 6–12	Abstainers	334/543	1.00 (0.86–1.16)	1.00 (0.82–1.22)
	Non-regular users	275/436	1.01 (0.87–1.16)	1.24 (1.00–1.50)
	Regular users	206/523	0.63 (0.53–0.74)	0.74 (0.61–0.91)
	*P* for trend		< 0.001	0.038
At age 13–18	Abstainers	326/515	1.00 (0.86–1.16)	1.00 (0.82–1.22)
	Non-regular users	343/617	0.86 (0.76–0.98)	0.96 (0.82–1.13)
	Regular users	146/370	0.61 (0.50–0.74)	0.68 (0.54–0.86)
	*P* for trend		< 0.001	0.019
At age 19–30	Abstainers	311/467	1.00 (0.86–1.17)	1.00 (0.82–1.22)
	Non-regular users	326/612	0.79 (0.69–0.90)	0.85 (0.72–1.00)
	Regular users	178/423	0.62 (0.52–0.74)	0.68 (0.55–0.84)
	*P* for trend		< 0.001	0.009
10 years before recruitment	Abstainers	316/513	1.00 (0.86–1.16)	1.00 (0.83–1.20)
	Non-regular users	305/535	0.93 (0.80–1.07)	0.97 (0.81–1.16)
	Regular users	194/454	0.70 (0.59–0.82)	0.72 (0.59–0.87)
	*P* for trend		< 0.001	0.019
**OTHER DAIRY PRODUCTS (ICE CREAM, YOGURT OR CHEESE), SERVINGS/MONTH**				
At age 13–18	Abstainers	205/301	1.00 (0.82–1.22)	1.00 (0.77–1.29)
	Non-regular users	402/836	0.71 (0.63–0.79)	0.85 (0.74–0.97)
	Regular users	208/365	0.84 (0.70–1.00)	0.92 (0.74–1.14)
	*P* for trend		0.29	0.73
At age 19–30	Abstainers	204/315	1.00 (0.82–1.21)	1.00 (0.76–1.31)
	Non-regular users	400/828	0.75 (0.67–0.84)	0.84 (0.73–0.96)
	Regular users	211/359	0.92 (0.77–1.10)	1.01 (0.81–1.25)
	*P* for trend		0.68	0.75
10 years before recruitment	Abstainers	202/328	1.00 (0.82–1.21)	1.00 (0.77–1.31)
	Non-regular users	399/821	0.80 (0.72–0.90)	0.88 (0.77–1.01)
	Regular users	214/353	1.01 (0.85–1.21)	1.05 (0.85–1.31)
	*P* for trend		0.79	0.59

† *Adjusted for sex, age (5-year group), socioeconomic status score (range: −1 [lowest] to 13 [highest], calculated by the subject's, and his/her father's and mother's education, housing type at age 10, personal income and household income), smoking and drinking status (never/ever), occupational hazards (never/ever), family history of cancer (none/NPC/other cancers), IgA against EBV viral capsid antigen VCA (EBV VCA-IgA, seronegative/seropositive), and total energy intake (residual method) at different life periods as appropriate*.

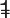
 *A flexible additive regression model with predictive mean matching incorporating data on the primary outcome (NPC cases vs. controls), exposure (milk or other dairy products intake) and other covariates included in the multivariable model was used to predict missing values of these factors*.

*All the risk estimates did not vary (P for sex interaction ranged 0.21–0.99) by sex*.

**Figure 1:**
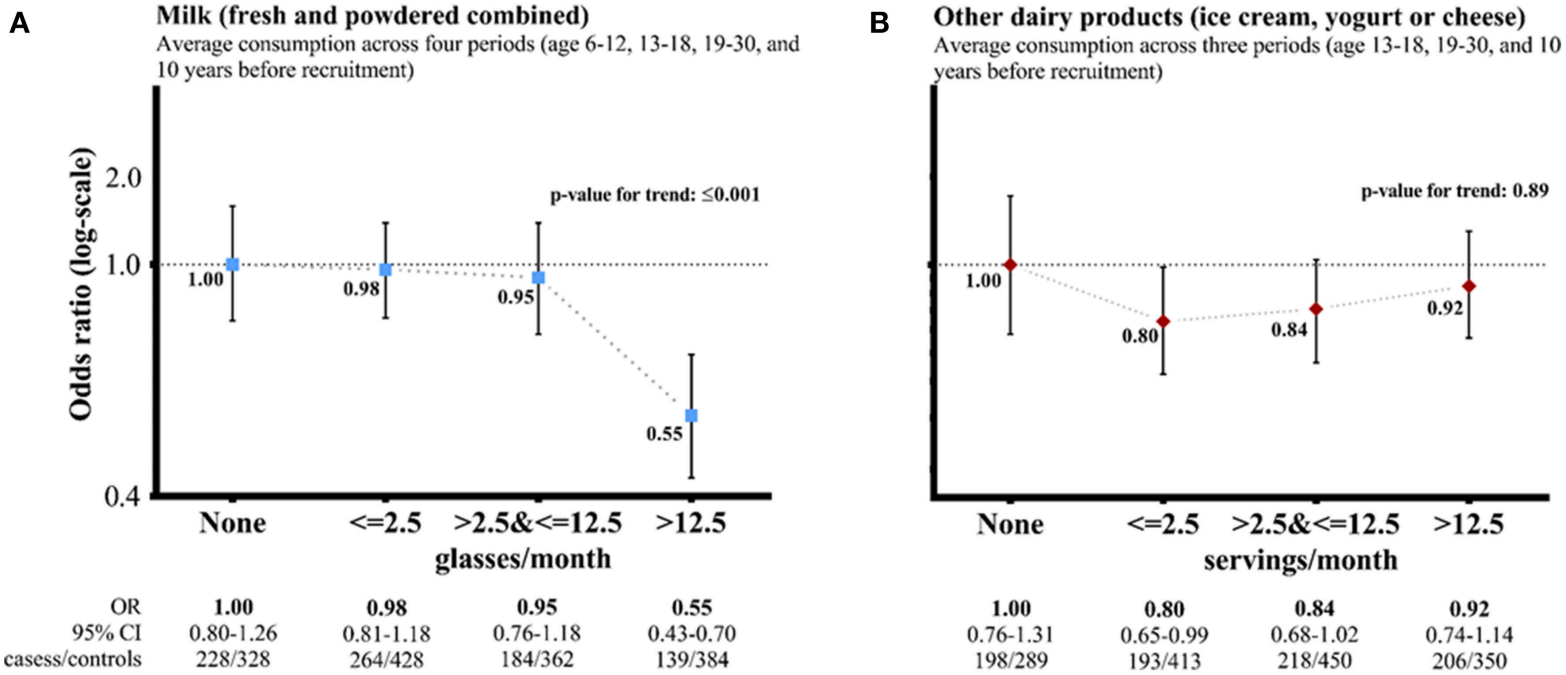
Dairy product consumption and risk of nasopharyngeal carcinoma (cases vs. controls)—adjusted† odds ratios (ORs, 95% confidence interval: CI) comparing average consumption across life periods (three categories) vs. none after multiple imputation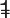. †Adjusted for sex, age (5-year group), socioeconomic status score, smoking and drinking status, exposure to any occupational hazard, family history of cancer, IgA against Epstein-Barr virus viral capsid antigen, and total energy intake at different life periods as appropriate. 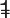 A flexible additive regression model with predictive mean matching incorporating data on the primary outcome (NPC cases vs. controls), exposure (milk or other dairy products) and other covariates included in the multivariate model was used to predict missing values of these factors. CI: group-specific confidence interval for the multivariable OR, reflecting the variance of the log risk in only that one group. The risk estimates did not vary by sex [*P* for interaction: 0.52 for **(A)** and 0.91 for **(B)**]. **(A)** Milk (fresh and powdered combined). **(B)** Other dairy products (ice cream, yogurt, or cheese).

Figure 1 shows the adjusted ORs for long-term average milk consumption of none, ≤2.5, >2.5 & ≤12.5, >12.5 glasses per month were, respectively, 1.00 (0.80–1.26), 0.98 (0.81–1.18), 0.95 (0.76–1.18), and 0.55 (0.43–0.70) (*P* for trend <0.001). For long-term average consumption of other dairy products, the adjusted ORs (95% CI) for none, ≤2.5, >2.5, and ≤12.5, >12.5 servings per month were, respectively, 1.00 (0.76–1.13), 0.80 (0.65–0.99), 0.84 (0.68–1.02), and 0.92 (0.74–1.14) (*P* for trend 0.89).

## Discussion

Consumption of milk but not other dairy products across life periods was associated with lower risks of NPC in Hong Kong. This is consistent with our ecological analysis of international data in which milk consumption was negatively correlated with the incidence of NPC. Results on the association between milk intake and risk of NPC are scarce. Previous case-control studies reported inconsistent results in different types of dairy products in different populations. One showed a negative association (5) and three showed no association in the East (6–8), and two showed a positive association in the West (9, 10). No prospective cohort studies and randomized controlled trials were found. One possible explanation for the negative association between milk intake and NPC is that milk contains estrogen that accounts for over 40% of estrone intake from foods (18). High levels of calcium, fat, protein and folate in milk may also have a role. These nutrients have been found to have anti-cancer effects through various pathways, like inducing apoptosis, anti-inflammation, anti-proliferation and DNA methylation. Further studies are needed to confirm these results.

The strength of the present study included: (1) being the largest series of NPC for investigating the associations with consumption of individual dairy products at different life periods, and (2) having reliable information on dairy consumption as shown in our reliability study (12). However, several limitations should be noted. First, despite adjusting for covariates, residual confounding cannot be excluded. Consumption of fresh fruits or vegetables was associated with NPC risk, but it has not been found to be associated with dairy intake. Indeed, dairy intake was not correlated with consumption of fresh fruits or vegetables in our analysis (data not shown). Therefore, consumption of fresh fruits or vegetables was not regarded as a potential confounder for the association between dairy intake and NPC risk in the present study. Nonetheless, further analysis yielded similar ORs (data not shown) after adjusting for consumption of fruits or vegetables. Mendelian randomization approach using the single nucleotide polymorphism (SNP) of LCT-13910 C/T is recommended, but a larger sample size is needed because of the relatively low frequency of the T allele that can digest milk (i.e., lower prevalence of lactase persistence) in our sample and in Chinese populations. Furthermore, as we used hospital-based control subjects, Berkson's bias might exist. Notably, the dairy consumption (milk and others) in our control group was lower than that in the general Hong Kong population, suggesting that our results might underestimate the protection of milk consumption against NPC. The external validity of our results might be limited because hospital-based controls were used. Further studies may recruit population-based controls or use other study designs (Mendelian randomization approach, prospective cohort studies, and randomized controlled trials) which can provide stronger evidence for causation. Another concern is potential information bias, especially as the range of dairy consumption was already limited, which made dose-response relations more difficult to detect. To limit any information bias, we designed and used a computer-assisted self-administered questionnaire to collect information on exposure of interest in the same way from both cases and controls. We also conducted a test-retest reliability study to assess recall error, and found that the questionnaire data of most NPC etiology factors of our NPC case-control had acceptable reliability (fair-to-substantial reliability), even for early life exposure (age 6–12 and 13–18) (12).

## Conclusions

Our data suggest milk intake may be a protective factor of NPC. Such protective association may be attributed to the estrogen, calcium, vitamin D (fortified), or folate in milk, but further research is needed for confirmation. Our result, if confirmed to be causal, has important implications for the consumption of dairy products and prevention of NPC in the East, where consumption of dairy products is generally low.

## Ethics Statement

The Institutional Review Board of the HKU/Hospital Authority HK West Cluster (UW 11-192), the HK East Cluster Research Ethnics Committee (HKEC-2012-043), the Research Ethics Committee of the Hospital Authority Kowloon Central/Kowloon East (KC/KE-13-0115/ER-2), the Research Ethics Committee of the Kowloon West Cluster [KW/EX-13-073(63-11)], and the NTW Cluster Clinical & Research Ethics Committee (NTWC/CREC/1239-13) approved the study. Informed consent was obtained from all individual subjects included in the study.

## Author Contributions

Z-MM, J-HL, and Y-HC designed and conducted the study in consultation with T-HL. S-YH is the guarantor for the paper. Z-MM analyzed the data, wrote the first draft, and has checked the accuracy and completeness of the references. All authors revised it critically for important intellectual content and contributed to final approval of the paper.

### Conflict of Interest Statement

The authors declare that the research was conducted in the absence of any commercial or financial relationships that could be construed as a potential conflict of interest.

## Abbreviations

NPC: nasopharyngeal carcinoma
OR: odds ratio
EBV: Epstein-Barr virus
VCA-IgA: IgA against EBV viral capsid antigen
SNP: single nucleotide polymorphism
rOD: relative optical density.
