# Supplementary material for: Milk Consumption Across Life Periods in Relation to Lower Risk of Nasopharyngeal Carcinoma: A Multicentre Case-Control Study
Source: Front Oncol. 2019 Apr 10;9:253. doi: 10.3389/fonc.2019.00253 (PMC6467951; doi:10.3389/fonc.2019.00253)
Supplement: Supplementary file 1 [file Table_1.DOCX]

| **Supplementary Table 1.** Adjusted odds ratios (ORs) and 95% confidence intervals (CIs) of nasopharyngeal carcinoma (cases versus controls) for dairy product consumption in 815 NPC cases and 1502 controls (available case analysis) | | | | | |
| --- | --- | --- | --- | --- | --- |
|  |  | **N**  **Cases/controls** |  |  | **Adjusted OR^†^ (95% CIs)** |
| **Milk (fresh and powdered combined), glasses/month** | | | |  |  |
| *At age 6-12* | Abstainers | 334/543 |  |  | 1.00 (0.71-1.42) |
|  | Non-regular users | 275/436 |  |  | 1.00 (0.74-1.36) |
|  | Regular users | 206/523 |  |  | **0.65 (0.46-0.91)** |
|  | P for trend |  |  |  | 0.08 |
|  |  |  |  |  |  |
| *At age 13-18* | Abstainers | 326/515 |  |  | 1.00 (0.71-1.40) |
|  | Non-regular users | 343/617 |  |  | 0.96 (0.73-1.27) |
|  | Regular users | 146/370 |  |  | **0.63 (0.43-0.93)** |
|  | P for trend |  |  |  | 0.10 |
|  |  |  |  |  |  |
| *At age 19-30* | Abstainers | 311/467 |  |  | 1.00 (0.71-1.41) |
|  | Non-regular users | 326/612 |  |  | 0.97 (0.73-1.29) |
|  | Regular users | 178/423 |  |  | **0.68 (0.48-0.98)** |
|  | P for trend |  |  |  | 0.14 |
|  |  |  |  |  |  |
| *10 years before recruitment* | Abstainers | 316/513 |  |  | 1.00 (0.73-1.36) |
|  | Non-regular users | 305/535 |  |  | 1.11 (0.82-1.51) |
|  | Regular users | 194/454 |  |  | 0.76 (0.54-1.08) |
|  | P for trend |  |  |  | 0.29 |
| *Average* milk intake across the above 4 *periods* | | | | | |
|  | None | 228/328 |  |  | 1.00 (0.66-1.52) |
|  | ≤2.5 | 264/428 |  |  | 0.88 (0.64-1.20) |
|  | >2.5 & ≤12.5 | 184/362 |  |  | 0.70 (0.49-1.01) |
|  | >12.5 | 139/384 |  |  | **0.58 (0.39-0.86)** |
|  | P for trend |  |  |  | 0.040 |
|  |  |  |  |  |  |
| **Other dairy products (ice cream, yogurt or cheese), servings/month** | | | | | |
| *At age 13-18* | Abstainers | 205/301 |  |  | 1.00 (0.52-1.91) |
|  | Non-regular users | 402/836 |  |  | **0.55 (0.43-0.71)** |
|  | Regular users | 208/365 |  |  | **0.49 (0.33-0.74)** |
|  | P for trend |  |  |  | 0.12 |
|  |  |  |  |  |  |
| *At age 19-30* | Abstainers | 204/315 |  |  | 1.00 (0.61-1.63) |
|  | Non-regular users | 400/828 |  |  | 0.96 (0.76-1.22) |
|  | Regular users | 211/359 |  |  | 0.77 (0.53-1.11) |
|  | P for trend |  |  |  | 0.33 |
|  |  |  |  |  |  |
| *10 years before recruitment* | Abstainers | 202/328 |  |  | 1.00 (0.62-1.62) |
|  | Non-regular users | 613/821 |  |  | 0.85 (0.67-1.07) |
|  | Regular users | 214/353 |  |  | 0.84 (0.57-1.22) |
|  | P for trend |  |  |  | 0.62 |
| *Average other dairy products intake across the above 3 periods* | | | | | |
|  | None | 198/289 |  |  | 1.00 (0.61-1.64) |
|  | ≤2.5 | 193/413 |  |  | 0.82 (0.58-1.15) |
|  | >2.5 & ≤12.5 | 218/450 |  |  | 0.99 (0.70-1.40) |
|  | >12.5 | 206/350 |  |  | 0.81 (0.55-1.19) |
|  | P for trend |  |  |  | 0.73 |
| † Adjusted for sex, age (5-year group), socioeconomic status score (range: -1 to 13, calculated by the subject's, and his/her father's and mother's education, personal income, household income and housing type at aged 10), smoking and drinking status (never/ever), exposure to occupational hazards (never/ever), family history of cancer (none/NPC/other cancers), IgA against Epstein-Barr virus viral capsid antigen (EBV VCA-IgA, seronegative/seropositive), and total energy intake (residual method) at different life periods as appropriate.  Subjects (cases/controls) with missing data in measurement of EBV VCA-IgA serostatus (296/478), smoking status (6/5), family history of cancer (124/111), and exposure to occupational hazards (131/147) were excluded in the multivariable analysis.  All the risk estimates did not vary (P for interaction ranged 0.19-0.93) by sex. | | | | | |

**Supplementary Part I. Methods of control recruitment**

Controls were recruited from the Departments of Medicine and Geriatrics, Surgery, Neurosurgery, Orthopaedics and Traumatology, and Ophthalmology in the same hospitals of the nasopharyngeal carcinoma (NPC) cases. New patients or referrals of a new health complaint within one year in the specialist outpatient clinics, or new inpatients admitted in the past three months who were frequency-matched by 5-year age group and sex to the cases were invited to participate. Three types of recruitment procedures for controls were implemented to suit the different settings:

1. Screening by clinical doctors or nurses

The doctors or nurses in the specific clinics screened for eligible subjects based on the criteria, and invited the eligible patients to participate in the study. The patients who agreed to participate were requested to sign the Informed Consent Form, and the bio-specimens were collected by the doctors or nurses. In most cases, our research assistants interviewed the patients immediately. If not, the patients were interviewed in their next appointment or hospital visit within one year.

2. Screening by research assistants

The research assistants with a medical background attended the specific clinics and screened the medical records to identify eligible subjects based on the criteria and invited those eligible to participate in the study. The patients who agreed to participate were requested to sign the Informed Consent Form. The patients were interviewed and the phlebotomists or our research assistants collected the bio-specimens in the hospitals. If not possible, the patients were interviewed and the bio-specimens were collected in the next appointment or hospital visit within one year.

3. New case list

The research assistants who were assigned to attend the clinics by the project coordinator retrieved the new case lists of the day (pulling lists) from the clinic registration office. The research assistants approached the eligible patients, invited them to participate in the study and sign the Informed Consent form. The patients were interviewed and the phlebotomists or our research assistants collected the bio-specimens in the hospitals.

**Inclusion criteria**

Broad categories of control diseases with the most common expected subcategories are listed below following the list of the AsiaLymph study (in International Statistical Classification of Diseases and Related Health Problems 9th Revision). Following the AsiaLymph guideline, we also specified that no more than 15% of controls had the same specific type of disease. A limited number of specific diagnoses were excluded from these broad groups, based on a known or suspected relation with vitamin D exposure, and immunological, infectious, and/or inflammatory aetiology. Specific conditions for inclusion were as follows:

**I. Acute injuries**

a. Dislocation (830-839)

b. Sprains and strains on joints and adjacent muscles (840-848)

c. Injury to blood vessels (900-904)

d. Contusion with intact skin surface (920-924)

e. Effects of foreign body entering through orifice (930-939)

**II. Diseases of the cardiorespiratory system**

Pneumothorax and air leak (512)

**III. Diseases of the digestive system**

a. Appendicitis (540-543)

b. Hernia of abdominal cavity (550-553)

c. Other diseases of intestines and peritoneum (560-569)

d. Other diseases of digestive system (574)

**IV. Diseases of the central nervous system and sense organs**

a. Cataract admitted for surgery (366)

b. Corneal injury/dendritic ulcer (700)

**V. Orthopaedic conditions clinically admitted for surgical operations**

a. Osteoarthritis for hip/knee replacement (715.15-715.16)

b. Intervertebral disc disorders (722)

c. Spondylolisthesis (756.12)

d. Acquired deformities of toe (735)

**VI. Other ambulatory surgical conditions**

a. Diseases of sebaceous glands (706)

b. Other disorders of skin and subcutaneous tissue (709)

**VII. Symptoms, signs, and ill-defined conditions**

a. Dizziness and giddiness (780.4)

b. Sleep disturbances (780.5)

c. Fever and other physiologic disturbances of temperature regulation (780.6)

d. Malaise and fatigue (780.7)

e. Generalised hyperhidrosis (780.8)

f. Other general symptoms (780.9)

g. Nonspecific findings on examination of blood (790)

h. Nonspecific findings on examination of urine (791)

**Exclusion criteria**

a) History of NPC

b) Subjects screened positive for suspected symptoms of NPC, including recent unilateral facial nerve palsy, tinnitus, unilateral hearing loss and epistaxis

c) Dementia

Of 1,777 eligible subjects approached, 1,512 completed the questionnaire and were included in the analysis (response rate: 85.1%). Among them, 1,053, 282 and 522 samples of blood, saliva and buccal swabs were collected, respectively.
